# Workability of mRNA Sequencing for Predicting Protein Abundance

**DOI:** 10.3390/genes14112065

**Published:** 2023-11-11

**Authors:** Elena A. Ponomarenko, George S. Krasnov, Olga I. Kiseleva, Polina A. Kryukova, Viktoriia A. Arzumanian, Georgii V. Dolgalev, Ekaterina V. Ilgisonis, Andrey V. Lisitsa, Ekaterina V. Poverennaya

**Affiliations:** 1 Institute of Biomedical Chemistry, Moscow 119121, Russia; 2 Engelhardt Institute of Molecular Biology, Russian Academy of Sciences, Moscow 119991, Russia; gskrasnov@mail.ru

**Keywords:** NGS, transcriptome, proteome, protein abundance, gene expression, mRNA-to-protein ratio, RNA-Seq, mass spectrometry

## Abstract

Transcriptomics methods (RNA-Seq, PCR) today are more routine and reproducible than proteomics methods, i.e., both mass spectrometry and immunochemical analysis. For this reason, most scientific studies are limited to assessing the level of mRNA content. At the same time, protein content (and its post-translational status) largely determines the cell’s state and behavior. Such a forced extrapolation of conclusions from the transcriptome to the proteome often seems unjustified. The ratios of “transcript-protein” pairs can vary by several orders of magnitude for different genes. As a rule, the correlation coefficient between transcriptome–proteome levels for different tissues does not exceed 0.3–0.5. Several characteristics determine the ratio between the content of mRNA and protein: among them, the rate of movement of the ribosome along the mRNA and the number of free ribosomes in the cell, the availability of tRNA, the secondary structure, and the localization of the transcript. The technical features of the experimental methods also significantly influence the levels of the transcript and protein of the corresponding gene on the outcome of the comparison. Given the above biological features and the performance of experimental and bioinformatic approaches, one may develop various models to predict proteomic profiles based on transcriptomic data. This review is devoted to the ability of RNA sequencing methods for protein abundance prediction.

## 1. The Attractiveness of Transcriptomic Methods for Assessing Gene Expression

Similar to the social sciences, the number of models, theories, and knowledge about mechanisms in molecular biology is significantly inferior to the total amount of experimental data obtained. These features distinguish molecular biology from mathematics or astronomy [1]. High-throughput methods of molecular profiling domesticated over the past two decades make it possible to assess gene expression at the transcriptomic and proteomic levels and screen for mutations and chromosomal rearrangements in a genome-wide mode. The profiling of epigenetic changes, such as DNA methylation and histone modifications, has also become routine.

Such data can be used for systematic study and subsequent multiomics modeling [2] of quantitative relationships between different “omics layers”: transcriptomic and epigenomic, and transcriptomic and proteomic [3]. For example, by studying the mRNA level, it is possible to reveal the mechanisms and patterns responsible for gene expression regulation at the epigenetic level by profiling histone marks and methylation of the corresponding DNA regions [4].

Data on gene expression at the mRNA and protein levels are used to identify the mechanisms and characteristics of the gene that determine the degree of dependence of the protein content on the abundance of the corresponding transcript [5].

Developing a high-precision bioinformatics tool, which utilizes transcript content data for protein profile prediction, seems attractive for several reasons. Firstly, the protein, not the transcript, is the final “effector” of the entire process of gene expression, starting from the transcription initiation. Differences at the proteomic rather than the transcriptomic level are primary when evaluating the influence of specific factors (e.g., drug therapy or development of pathology) on cellular processes. Secondly, the ratios between gene expression products at the transcript and protein level can drastically (by several orders of magnitude) differ between distinct genes. Therefore, transferring findings drawn from transcriptomic data to the proteome level can often lead to incorrect conclusions [6]. The proteomic picture is additionally complicated by the presence of post-translational modifications (PTMs), which may completely change the activity and functions of the protein. Thirdly, transcriptomic profiling methods (RNA-Seq, microarrays, PCR) have significantly higher performance, convenience, and sensitivity compared to proteomic studies. The indicated advantages relate to panoramic (LC-MS/MALDI-TOF) and targeted (MRM and SRM) mass spectrometric approaches, as well as immunochemical methods [7]. On average, transcriptomic analysis (particularly RNA-seq) provides information on a significantly larger number of genes than proteomic analysis [8,9]. Thus, it is possible to quantify the expression level of ca. 10–18 thousand human mRNAs [10,11,12]. For comparison, routine panoramic mass spectrometric tissue analysis provides information about 3–5 thousand proteins [13].

Another advantage of RNA-Seq is the ability to provide comprehensive information about the complete transcript sequence, including point mutations, insertions, and deletions, and simultaneously identify cases of alternative splicing [14], including de novo analysis. The situation is entirely different with proteomic methods. In mass spectrometric analysis, protein detection and quantification are carried out mainly by analyzing small proteotypic peptides. In panoramic mode, non-specific amino acid substitutions or other protein sequence changes are usually not considered. The “Brute force” approach, which involves the enumeration of all possible aberrations, often expands the search space, increasing the fraction of false positives [15,16,17]. The same can be said about immunochemical methods: introducing a few substitutions in the protein sequence can significantly affect the affinity-binding constants of a protein and the corresponding antibody [18].

A deep and quantitative assessment of gene expression at the level of transcripts is much easier to perform than at the level of proteins [19]. The main reasons for the insufficient effectiveness of proteomic approaches are:

- the absence of an analogue of direct amplification of protein sequences, in contrast to genes and transcripts [20];
- the high diversity of proteinogenic amino acids (23 proteinogenic blocks and modified amino acids [21]) compared to the four-letter nucleotide composition of RNAs (even considering possible PTMs);
- the lack of high-specific enzyme selection to cleave protein sequences (compared to a rich set of polymerases, DNases, RNases, and ligases).

In mass spectrometric proteomic analysis, non-standard technical solutions can increase the number of identifications. Nevertheless, such solutions make the analysis time-consuming and inapplicable for the routine assessment of protein profiles. One example is proteolysis by multiple proteases [22] or multidimensional fractionation (2DE, 2D-LC). It was shown that two-dimensional alkaline fractionation doubled the coverage of protein sequences (from 23% to 54%) in a shotgun MS experiment [23].

The high value of knowledge about proteins coupled with imperfect proteomic technologies makes it essential to develop methods for predicting proteomic profiles based on transcriptomic data for a cell or tissue, and to study the patterns connecting the transcriptome and proteome.

## 2. Key Points of Transcriptome-to-Proteome Research

The central dogma of molecular biology links information flows between DNA, RNA, and protein [24]. Although the DNA sequence generally determines the sequence of transcribed mRNA, which defines the arrangement of amino acids in a protein, there is no trivial relationship between the abundance of the transcript and the corresponding protein. Only indirect relationships between the abundance of transcripts and proteins were revealed for model objects. However, in principle, there is a possibility to predict the protein content based on genomic and transcriptome data [25]. It has been shown that the amount of protein is not a linear function of the amount of the corresponding mRNA [26]. The level of mRNA by itself was also demonstrated to be insufficient to predict protein representation and explain the relationship between genotype and phenotype [27].

**Figure 1:**
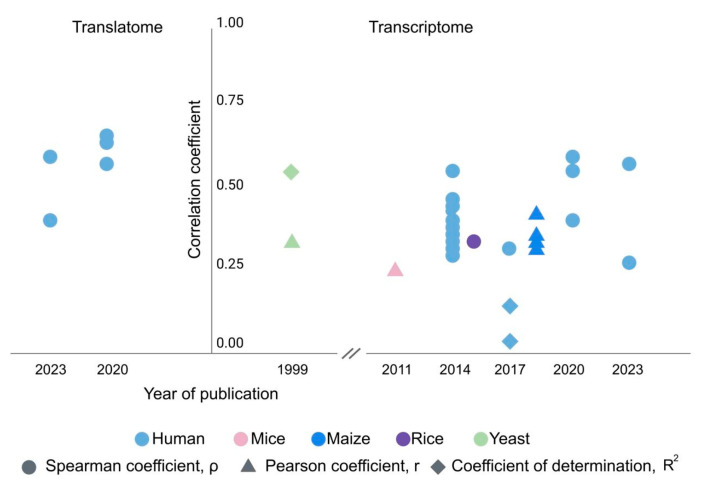
Timeline of mRNA-to-protein correlation coefficient based on transcriptome and translatome data.

Initially, based on the data on individual genes, it was believed [28] that the levels of mRNA and protein strongly correlate, but the development of large-scale and high-throughput transcriptomic and proteomic methods refuted this hypothesis (see Figure 1). Such global analyses have shown that transcripts with similar abundance levels can have corresponding proteins with widely varying concentrations. Some pioneering works [29], dedicated to the investigation of the relationship between the transcriptome and the proteome of *S. cerevisiae*, a popular object for developing predictive models, revealed that the protein content could vary as much as 20-fold at the same levels of corresponding RNAs.

Further investigations have shown that the coefficient of determination *R*^2^ = 0.58 between mRNA and protein levels can appear after log transformation of the abundance values, which often yields a Gaussian distribution of abundance data [30].

Another paper shows a considerably higher correlation between the number of mRNA copies and the absolute number of protein copies at *R*^2^ = 0.73 [31] (Figure 1). However, gene expression values and protein concentrations were obtained by at least two technologies and then averaged [31]. Thus, averaging across technologies removes technology-specific errors [31].

Other studies using direct quantitative estimates demonstrate significantly more modest correlation coefficients between the levels of transcripts and proteins. This conclusion was made in the study [32] of the transcriptome (RNA-Seq) and proteome (LC-MS) of liver tissues of 100 mice descending from representatives of inbred lines. The abundances of 5000 peptides and 22,000 transcripts were evaluated, from which around 500 proteins and 7000 mRNAs with the most accurate measurement results were selected for correlation analysis [32]. The study indicated a pronounced positive correlation between the levels of proteins and transcripts (with an averaged Pearson correlation coefficient of 0.27) (Figure 1).

In other works on plants (sowing rice and maize [33,34]), very weak correlations between transcriptome and proteome were also observed, both indicating a Pearson coefficient of linear relationship lower than 0.4 (Figure 1).

Various approaches for deriving transcriptomic and proteomic data in these studies represent the existing prosperity of methods. For example, the first study explored the quantitative relationship between the abundances of mRNA, derived from RNA-seq (levels reported in RPKM), and protein, gained via MS label-free relative quantification (intensity-based absolute quantification, or iBAQ) [34]. The pairs of values presented transcriptomic and proteomic profiles en masse to illustrate tissue-specific patterns of gene expression. The second study aimed the investigation of changes in gene expression during the development and specification of leaf vascular systems, and took, as units, differentially expressed proteins (DEPs), derived from MS isobaric group labeling for relative and absolute quantification (iTRAQ), and genes (DEGs) [33]. Transcript abundances were acquired through newly introduced high-throughput tag-sequencing for digital gene expression (DGE) analysis.

Interestingly, the authors of [34] noted that the expression of critical genes responsible for the morphology and function of maize leaf tissues was regulated precisely at the transcription level, depending on time and localization in the plant. In other cases, a low correlation indicates divergence in regulation on transcriptional and translational levels.

Meta-analysis and the creation of combined transcriptomic and proteomic datasets with subsequent correlation analysis were used in the study conducted on yeast [28]; it was shown that the correlation coefficient calculated for the combined data set was higher than the corresponding values calculated for individual data sets. In another work, non-linear optimization was used to estimate undetectable *D. vulgaris* proteins based on mRNA level data [35]. The method is based on the maximization of the objective function that describes the relationship between the transcriptomic and proteomic networks without considering network changes over time.

## 3. Gene-Centric Approach: The mRNA-Protein Ratio Varies Greatly between Different Genes but Is Conserved in Different Tissues and Cell Types

Several scientific groups have found that the ratio of transcripts to proteins for corresponding genes is relatively stable in various cell lines [36] and tissues [37] (Figure 1). This observation gave rise to the assumption that the protein content in any tissue can be predicted by the amount of mRNA [37,38]. Various factors can be responsible for the ratio of protein and mRNA levels; among them are:

(a) constant gene-specific and context-independent (i.e., codon selection, secondary structures of transcript and mRNA, protein tertiary structure, molecular weight, tRNA repertoire);
(b) depending on the specific state of the cell (the number of available ribosomes and translation initiation factors, the availability of tRNA, the rate of protein degradation).

The factors listed above can be included in a single regression model that makes it possible to link the mRNA level with the content of the encoded protein [39]. This approach was used by Frederic Edfors et al. [3]. The mRNA-to-protein ratio (RTP) for a particular gene is relatively constant between different tissues or cell lines. Thus, RTP is determined to a greater extent by the constant parameters of the gene or protein itself (ribosome advancement rate, tRNA preference, mRNA secondary structure, protein stability), rather than by a dynamic biological context that depends on the type of cell or tissue, i.e., the specific state of the cell [40]. The RTP values themselves between different genes can vary with a range of several orders of magnitude. This distribution differs between groups of genes involved in different biological processes and is associated with protein size in inverse dependence. In general, while the Pearson correlation coefficient between mRNA-protein levels estimated by the authors averaged 0.6–0.7, such a correction for RTP made it possible to increase it to values exceeding 0.9 [3,41].

According to other researchers, the value of 0.9 is overestimated: the high variability between genes in different tissues (3 × 10^6^), as well as between different genes in the same tissue (1.7 × 10^5^), creates a high correlation for observed and predicted protein levels; even if for individual genes, this correlation is weak [38]. This effect is similar to Simpson’s paradox: if two groups show the same trend, then the wrong combination of these data can change the direction of the relationship. To prove this assumption, Franks et al. [42] decided to reanalyze the raw data from the previous studies on human tissues [3,37,43]. Their results suggest a poor correlation (R = 0.33 for all measured mRNA across 12 tissues) between protein and mRNA contents for the same genes across various tissues, which they attribute to the extensive post-transcriptional regulation (Figure 1).

Interesting results of the group of Schwanhäusser et al. [5] shed light on the ratio of the cellular content of mRNA and protein. In 2011, the analysis of the content and turnover levels of mRNA and proteins corresponding to 5000 genes demonstrated that the half-lives of mRNA and protein do not correlate, in contrast to the levels of mRNA and protein levels. Moreover, the lifetime of proteins and mRNA is associated with the biological process in which the gene is involved.

In addition, it was shown that the cellular protein content is controlled mainly at the translation level [5]. Nevertheless, more than 85% of the variability in protein copy numbers (between samples, cells, or tissues) is determined by variability at the mRNA level when taking into account the gene-specific rate of translation and degradation, i.e., mRNA concentration remains a crucial factor for predicting protein levels [5].

For the first time, gene-centric coefficients of the ratio between mRNA and protein levels were obtained by Futcher et al. back in 1999 for a limited set of genes [30]. For example, on average, a single yeast (*S. cerevisiae*) cell contained only 54 mRNA molecules encoding actin (ACT1) and 160,000–205,000 actin protein molecules. For cytosolic aldehyde dehydrogenase (ALD6), this ratio was even more dramatic: three mRNA molecules and 160,000–180,000 protein molecules. Hence, the authors derived the following conclusion: the average doubling time of yeast colony is ca. 2 h, and one actin mRNA molecule accounts for ca. 4000 protein molecules. Therefore, the translation of each transcript is initiated approximately every 2 s. In turn, this means that if the average mRNA carries ten ribosomes involved in translation, then each ribosome completes translation in 20 s, assuming that the average protein has about 450 amino acid residues. Thus, it can be concluded that yeast is characterized by translating about 20 amino acids per second [30]. Interestingly, the same indicator for mammals is lower and amounts to approximately three to eight amino acids per second [44]. Following these observations, we can expect intertaxon variability in the correlation between mRNA and protein levels for different organisms.

Regarding relationships in the dynamics of protein and mRNA, the work of Cheng et al. [27] is of interest. The authors showed that in response to cellular stress (exposure to dithiothreitol), mRNA content increases abruptly, followed by a gradual increase in the concentration of encoded proteins (within several hours). At the same time, the protein content increases by a much greater amplitude than the mRNA level. This emphasizes that protein content is regulated both at the level of gene transcription and mRNA translation [27]. The authors of the study used a previously developed algorithm for isolating (deconvolution) the features of the regulation of protein and mRNA synthesis based on data on temporal profiling of the levels of transcripts and encoded proteins (PECA) [45]. Unlike experimental procedures such as SILAC [46], the PECA algorithm does not separate the intensities of protein synthesis and degradation.

One of the most striking works in the analysis of the relationship between transcriptomic and proteomic parameters is the study by Matthias Wilhelm, published in 2014 [37]. This is a landmark work on the drafting of the human proteome. The authors evaluated the gene-specific translation rates by median values in tissues [38]. A model for the quantitative relationship between protein level (iBAQ) and mRNA (RNA-Seq) was built based on expression profiles for 12 human tissues. The resulting Spearman correlation coefficient between the measured levels of protein and mRNA (from R = 0.41 for the thyroid gland to R = 0.55 for the kidney) turned out to be lower than previously shown for cell lines [37] (Figure 1). In addition, the authors showed that cell lines indeed inherit the main features of the expression profiles of both genes and proteins from their progenitor tissues. Still, original tissues have greater variability in expression than the cell lines derived from them [37].

The most complete model based on many factors and linking the transcriptomic and proteomic levels of the organization of living systems is described in [47]. This work is a bioinformatics processing of data obtained in a large-scale study [10]. In the proposed model, the authors obtained mRNA concentration values by RNA sequencing of 29 healthy human tissues (in total, more than 11.5 thousand protein-coding genes were analyzed at the transcriptome and proteome levels).

The resulting kinetic model included the following parameters: the level of mRNA concentration, the level of protein concentration, the number of free ribosomes in the cell, the rate of translation, and the half-life of proteins and mRNA. The result of the work is the predicted level of TPR (the ratio of mRNA to the level of the corresponding protein) for 11.5 thousand genes. It was shown that the variability of this indicator between genes in one tissue significantly exceeds the variability between the same gene in different tissues, which proves the previous statements about the gene-specificity of TPR.

## 4. Regulation of Gene Expression

Gene expression is regulated at multiple levels, including transcription, translation, and post-translational modification. These processes encompass RNA synthesis (which includes epigenetic and transcriptional regulation), RNA degradation, protein synthesis (or translational control), and protein degradation. Collectively, they determine the protein pool in a cell, as outlined by the central dogma of molecular biology. While protein synthesis and degradation generally play a more significant role than RNA synthesis and degradation, all these processes are incorporated into predictive models.

The protein level is related to the level of the coding transcript by several “constant” factors determined by the mRNA sequence, protein stability, etc. That is why the difference in mRNA-protein levels undergoes more significant variability between genes (within the same tissue or cell line) than between different tissues for the same gene [10]. However, relying on “constant” coefficients alone is insufficient to predict protein levels [41]. The authors [41] showed that the concentration of the CD81 protein (transmembrane protein mediating signal transduction and developing complex with integrins) in different tissues varies by two orders of magnitude at the same mRNA concentrations. At the same time, the concentration of MEF2D (transcription factor) mRNA varies up to tenfold at the same protein concentrations.

Thus, RNA/protein synthesis and degradation depend on “constant” factors and the ones that depend on the state of the cell. So, these factors—DNA methylation [48], mRNA modifications [49], changes in the histone code [50], (de)condensation of chromatin [51], binding of various transcription factors [52], alternative splicing [53], the ratio of exon length to the total length of the transcribed region [54], and polyadenylation processes [55,56]—can be identified using special technology of DNA or RNA sequencing and bioinformatics algorithms.

The importance of integrating various data for all ways of gene expression regulation can be illustrated by the example of the influence of the composition of transcript isoforms encoding the protein on the rate of protein translation and degradation rate. Floor and Doudna [57] performed dissemination on the influence of transcript structure on protein translation efficiency. Their analysis strategy, Transcript Isoforms in Polysomes sequencing (TrIP-seq), combined polysome profiling with global gene expression analysis. TrIP-seq reveals transcript-isoform-specific translation patterns. In line with the previous findings, the authors confirmed that transcripts that contain the same ORF but different UTRs could have strikingly different translation rates. A few years later, Salovska et al. [58] showed that individual protein isoforms of the same genes can have different degradation rates, significantly impacting the protein abundance levels.

The epitranscriptome presents a complex landscape of post-transcriptional modifications that can significantly impact protein expression levels [49]. One prominent example is the m6A modification, which has been shown to both enhance [59], and inhibit translation processes [60]. Traditional methods for analyzing m6A modification sites in transcripts include m6A-seq, MeRIP-seq, miClip, and m6A-CLIP [61]. Alternatively, direct RNA sequencing performed on Oxford Nanopore technology can be processed through bioinformatic tools to identify m6A modification sites. Understanding the effects of such modifications on protein expression levels is critical for advancing our knowledge of cellular processes and disease mechanisms [62].

Another mechanism of translation regulation is RNA-interference, caused by microRNA (miRNA) and small interfering RNA (siRNA). There are different functions of gene regulation for these types of RNAs and one of them is to inhibit protein translation of the target mRNA [63,64,65,66]. For example, the expression of several microRNAs leads to translation inhibition, which in turn leads to the development of fibrosis [67]. Small RNA-seq (sRNA-seq), also called microRNA-seq (miRNA-seq), allows identifying miRNA and siRNA as a bulk mode [68], so as single-cell analysis [69].

In the context of translation regulation, it is also worth mentioning QTLs, which can be detected by DNA sequencing. So, for 30% of the 199 unique protein approved by the FDA as biomarkers, pQTLs were found [70]. The pQTLs explain the reports of different basic contents of these biomarkers in people of different races [71].

## 5. Translatome

Previously, it has been elucidated that the abundance of proteins results from a complex cascade of gene expression, which is meticulously regulated at each stage. The impact of regulatory factors extends beyond generating a specific set of transcripts. Numerous studies suggest that merely 40% of protein variability is determined at the transcriptome level [5,19].

Presumably, the major part of regulation comes from the process of translation [72]. Thus, the true predictive power remains in the translates, which refers to the functionally activated set of mRNAs undergoing protein synthesis [73].

Indeed, genes are transcribed and translated with varying intensity depending on the cell’s current needs and context [74]. This is true for both complex cellular differentiation processes and maintaining homeostasis [75]. For example, for several genes involved in cellular development, an energetically unfavorable combination of active transcription and translational repression mechanisms (translational control) mediated by RNA-binding proteins has been observed [76]. The correlation will be negative until the cell requires the synthesis of that particular protein [77].

There are compensatory mechanisms in the evolution of genetic expression that maintain a stable protein composition [78]. Translational control does not always “sustain” the direction of the transcriptional one [79]. For example, post-transcriptional level mutations can restore the level of protein synthesis that was reduced due to transcriptional regulation [80]. From this perspective, the translatome is more significant as it is closer to the proteome in the chain of gene expression processes.

A meaningful study of this level of genetic expression would have been impossible without the development of sophisticated approaches. The earliest method, polysome profiling, was developed back in the 1960s. It is based on the assumption that ribosomes, the largest macromolecular complexes in most cells, will sediment in a sucrose gradient faster than other organelles [72].

The revolutionary method named ribosome profiling (Ribo-seq) implements deep sequencing of mRNA fragments, protected from the RNases’ “attack” by the binding of ribosome complexes, and therefore called ‘footprints’ [81]. As the control sample proceeds traditional mRNA sequencing, on the output researchers gain an overview of how ribosomes move along transcripts. This is characterized by the translation efficiency (TE) rate [81], which gives the idea of ribosome occupancy per mRNA. It can vary widely for different transcripts of life stages [82]. Meanwhile, as indicated in a study on drosophila as a model object in steady state (via TRAP methodology), most abundant transcripts are not those carrying the main amount of ribosomes [83].

Another, already noted approach is to tag polysomes via protein fusion to gain specific affinity (ribosome affinity purification (RAP) or translating RAP (TRAP)) [68]. Ribosome profiling is characterized by short readouts (depending on the chosen RNase [84,85]), which makes this method sensitive to noise. However, today it is the most common method for translatome research. With the ability of this method to provide information on the “location” of ribosomes down to the nucleotide level, it can be used to study and characterize both canonical and non-canonical ORFs [86]. The discovery of these ORFs significantly expands the number of coding sequences (CDS).

A number of studies have compared the correlation between the translatome and the transcriptome with the proteome in lower eukaryotes [82,87]. It has been generally shown that the translatome correlates more with the proteome than the transcriptome. Thus, the Spearman correlation between the proteome and the translatome (Ribo-seq) in normal *S. cerevisiae* cells was 0.77 [87]. Contrariwise, the correlation between the proteome and transcriptome was 0.46.

In contrast to what has been discussed earlier for transcriptome, translatome is characterized by far less divergence in gene expression between tissues [79]. It indicates the evidence of post-transcriptional buffering [88].

In the study conducted on human tissues, the correlation between the translatome and proteome was measured for 9642 genes present in every sample. For organs such as the brain, liver and testis, Spearman’s correlation coefficient yielded values ρ = 0.65, 0.69, and 0.60, respectively. In comparison, the corresponding correlation ratios between the transcriptome and proteome were ρ = 0.57, 0.61, and 0.42 [10,89].

As previously mentioned, a high correlation is associated with the contribution of post-transcriptional regulation. For instance, in an experiment on a mouse cell line, it is independently implemented for 20% of differentially expressed genes [79].

The primary mechanisms of translational control are specifically directed towards regulating initiation [90], although they can also be applied to post-initiation steps, elongation, and termination of translation.

Interesting findings were obtained in an experiment focused on the effects of hypoxic stress on cardiomyocytes [91]. It appeared that with the increase in the intensity of the stress stimulus, cells may change their preference of a strategy aimed at enhancing the expression of specific genes. For example, short-term hypoxia results in changes at the translatome level by increasing ribosome recruitment on mRNA (upregulation via binding of NCBP3 protein to 5′-UTR). Indeed, enhancing translation is the fastest way to increase the representation of necessary proteins within a cell. Translational load focuses on genes associated with the HIF-1 signaling cascade. This cascade induces rapid changes in cellular physiology [92]. On the contrary, prolonged hypoxia leads to enhanced synthesis of a specific subset of transcripts, involving genes associated with autophagy, apoptosis, and cell proliferation. These are the processes associated with long-term effects or novel cell functions, in which implementation requires the activation of previously silent genes. Post-translational regulation adjusts pre-existing protein composition to a new cellular context [26].

Translational control also takes precedence when regulation at the transcriptional level is not manifested. This is the case in developmental processes [93]. Another curious example is the case of trypanosomatids’ differentiation into an infective form, completely regulated post-transcriptionally [82]. There is evidence of changes in the translatome during disease or malignant transformation of an organ [94].

Translatomics and transcriptomics in this paradigm should complement each other. The perfect model for protein abundance prediction probably considers the strength of their respective contributions.

## 6. Single-Cell Transcriptomics–Proteomics

The previous discussion was dedicated only to insights derived from studies of cell populations at the bulk level. However, a question arises of how well the observed relationship between the transcriptome and the proteome carries over to the level of individual cells. Thanks to the recent advancements in the field of single-cell transcriptomics, the picture of the regulation of gene expression at the level of RNA is starting to become clear [95]. First of all, it is now well established that gene expression in individual living cells occurs in the form of stochastic bursts, also called transcriptional bursts [96]. This phenomenon introduces a significant amount of variation in the cellular levels of individual mRNAs, which can be difficult to separate from other regulated factors affecting gene regulation, such as the activity of transcriptional factors, but approaches to mitigating this problem are being developed [97]. Additional challenges for single-cell-level quantitative analysis include the detection of products of low-expressed genes [98], and the accurate quantification of transcript isoforms [99]. Despite these and other challenges [100], single-cell transcriptomics is progressing rapidly. It has already provided high-quality portraits of gene expression for various complex cell populations such as tissues, revealing important details about vital biological processes [101,102,103,104].

The robustness of single-cell transcriptomics techniques is largely attributed to the availability of methods for amplifying individual DNA molecules, which, as mentioned at the beginning of the article, is currently unavailable for proteomics. Despite this, approaches to adapt mass spectrometry-based identification of proteins for single cells are underway [105]. As these methods feature significant downscaling, most of the published methods of this category so far require drastic modifications to the typical mass spectrometry protocol, and, as a consequence, the capability of such methods currently lags behind the respective transcriptomics techniques [106]. Still, as these approaches are constantly refined and updated [107], we are now starting to see the first published global comparisons of transcriptomes and proteomes for single cells. In a recent paper by Brunner et al., a novel single-cell mass spectrometry workflow (T-SCP) was separately complemented by two established single-cell RNA-Seq techniques (SMARTSeq2 and Drop-Seq) to investigate the proteogenomics of the HeLa cell line, and products of 1672 genes were detected by all three methods [108]. Surprisingly, HeLa cells demonstrated a much better correlation between each other at the proteome, rather than the transcriptome level, once again highlighting the aforementioned highly stochastic nature of gene expression in individual cells. To investigate transcriptome-to-proteome correlation, the coefficients of variation for shared genes between all data sets were compared, revealing that variation at the gene level did not correlate well between the transcriptome and the proteome, in line with the insights from the bulk-level analysis. One more study, currently available as a preprint, reports the development of a protocol for the parallel measurement of transcriptomes and proteomes in single cells using nanodroplet splitting. After successfully applying this protocol to C10 and SVEC cells, a subsequent correlation analysis between transcript and protein abundances produced Pearson’s correlation coefficients ranging from 0.31 to 0.56, with values once again similar to previous analyses. In conclusion, even though transcriptional regulation is significantly different at the level of single cells than at the bulk level, presently available data suggests that the correlation between the transcriptome and the proteome remains at the same, rather modest levels.

Parallel profiling at several omics levels of proteins and nucleic acids isolated from a single cell eliminates the problem of genotypic or phenotypic heterogeneity of the bulk sample. The transition from an “average” cell to a “single” cell is an unprecedented opportunity to unambiguously determine the correlation between the transcriptome and the proteome and, subsequently, with the phenotype [109].

To understand how a single cell relates to its averaged portrait obtained from cells in bulk is a difficult but manageable task [100]. In recent years, several methods have been developed to robustly and safely isolate individual cells and quantify their content (i.e., laser capture microdissection [110], robotic micromanipulation [111], microfluidics coupled with RNA-Seq or proteomics techniques [112] and fluorescent methods—fluorescence-activated cell sorting (FACS) [113] or fluorescence microscopy [114]). One of the most straightforward and elegant approaches is via a proximity-based assay, such as the proximity ligation assay (PLA) or proximity extension assay (PEA) [115]. These assays allow amplifying the signal from a single protein (or a pair of interacting proteins) with a PCR-like mechanism, greatly increasing the sensitivity. Briefly, the PEA features two protein-specific antibodies, which are tagged with complementary oligonucleotides. When both of these antibodies interact with the same protein molecule, and are thus in proximity (hence the method name), complementary oligonucleotides hybridize, creating a site that can be amplified with specifically designed primers, thus increasing the copy number of this nucleotide segment, which can be detected via RT-PCR or NGS methods.

## 7. Conclusions

While the genome contains information about liability to disease, and the transcriptome can be reliably quantified, only the accurate measurements of the proteome and the metabolome can most fully reveal the current state of the organism, i.e., create a digital profile of the organism for a particular time point [116]. Thus, there is a need to develop a high-quality model of the relationship between transcriptomic and proteomic data since the current capabilities of RNA-seq are much superior to proteomic methods. Assessing the correlation between different layers of information transfer inside the cell is important for multi-omics analyses, allowing for addressing the missing data or even fully predicting one omics layer from another [117].

The knowledge of transcript abundance is insufficient for predicting protein abundance due to many factors. A cohort of large-scale investigations aimed at comparing the levels of transcripts and proteins at a genome-wide scale have led to the identification of several key factors affecting the quantitative relationship between the transcriptome and the proteome. These include the rate of translation (which in turn depends on the mRNA sequence), the activity of the regulatory elements (miRNAs and other non-coding RNAs), the relative availability of ribosomes, and protein degradation rate. Notably, these factors are highly dynamic and often specific for a particular protein, which makes the development of global predictive models extremely challenging.

However, current capabilities of nucleic acid sequencing methods provide a wide scope for studying the processes of expression regulation. Taking into account a number of factors, the modifications of DNA and RNA nucleotides, the level of expression of transcripts and small RNAs, and the level of translation, etc., obtained through specialized sequencing approaches, allows us to adjust the mRNA-protein model, achieving high prediction rates.

## Data Availability

Not applicable.
